# Reforestation Sites Show Similar and Nested AMF Communities to an Adjacent Pristine Forest in a Tropical Mountain Area of South Ecuador

**DOI:** 10.1371/journal.pone.0063524

**Published:** 2013-05-06

**Authors:** Ingeborg Haug, Sabrina Setaro, Juan Pablo Suárez

**Affiliations:** 1 Institute of Evolution and Ecology, Plant Evolutionary Ecology, University of Tübingen, Tübingen, Germany; 2 Department of Biology, Wake Forest University, Winston-Salem, North Carolina, United States of America; 3 Departamento de Ciencias Naturales, Universidad Técnica Particular de Loja, Loja, Ecuador; Nanjing Agricultural University, China

## Abstract

Arbuscular mycorrhizae are important for growth and survival of tropical trees. We studied the community of arbuscular mycorrhizal fungi in a tropical mountain rain forest and in neighbouring reforestation plots in the area of Reserva Biológica San Francisco (South Ecuador). The arbuscular mycorrhizal fungi were analysed with molecular methods sequencing part of the 18 S rDNA. The sequences were classified as Operational Taxonomic Units (OTUs). We found high fungal species richness with OTUs belonging to Glomerales, Diversisporales and Archaeosporales. Despite intensive sampling, the rarefaction curves are still unsaturated for the pristine forest and the reforestation plots. The communities consisted of few frequent and many rare species. No specific interactions are recognizable. The plant individuals are associated with one to ten arbuscular mycorrhizal fungi and mostly with one to four. The fungal compositions associated with single plant individuals show a great variability and variety within one plant species. Planted and naturally occurring plants show high similarities in their fungal communities. Pristine forest and reforestation plots showed similar richness, similar diversity and a significantly nested structure of plant-AMF community. The results indicate that small-scale fragmentation presently found in this area has not destroyed the natural AMF community, at least yet. Thus, the regeneration potential of natural forest vegetation at the tested sites is not inhibited by a lack of appropriate mycobionts.

## Introduction

How to maintain biodiversity increasingly draws attention in connection with ecological devastation and climate change. A special focus is put on highly complex and species-rich ecosystems, the so-called hotspots of biodiversity. One example of a biodiversity hotspot is the tropical mountain rain forest in southern Ecuador [1], [2], [3]. These forests harbour tens of thousands of species that interact with each other. It is impossible to understand an ecosystem without knowing the composition and interactions of its community. Even though arbuscular mycorrhizal fungi (AMF) play an important role in the nutrition of nearly all plants, there are only few molecular studies about AMF in tropical forests [4], [5]. Composition of AMF and their interactions with the diverse tropical plant community are not well known [6]. Theories on biodiversity and ecosystem stability of multispecies assemblages only recently integrated mutualistic interactions [7]. During the last decade, network theories were applied to analyse species-rich, mutualistic interactions and emphasized plant-animal assemblages such as pollinator and seed disperser webs [8], [9], [10], [11]. Analyses revealed general features in network architecture of fundamental importance for community formation, biodiversity persistence and stability of ecosystems [8], [12]. In contrast to antagonistic interactions arranged in compartments, mutualistic interactions show a nested structure: rare species tend to interact with a subset of species that generalists interact with [13]. Nestedness reduces effective interspecific competition, enhances the number of coexisting species – especially rare species - and increases robustness against loss of species [8], [12]. Studies on network architecture of mycorrhizae have so far only addressed one orchid genus in Europe [14], epiphytic versus terrestrial orchids on La Réunion [15] and the structure of two communities of plant-arbuscular mycorrhizal fungi [16], [17]. Preliminary network analyses for our study site will be presented in [18], but with a limited data set. Except for the orchid-mycorrhizal networks on La Réunion, the mycorrhizal associations showed significantly nested assemblages.

In this study, we compiled a broad, culture independent molecular data set of AMF mycobionts in a tropical area by sampling mycorrhizae of seedlings and mature trees from the pristine forest. Additionally we sampled mycorrhizae of grasses, shrubs, trees and planted indigenous tree seedlings on neighbouring reforestation sites. Preliminary results were published in [19], showing that diversity at the reforestation plots was unexpectedly high and comparable to the pristine forest. However, AMF community composition differed. For the current study, methodology was improved and the sampling expanded. In consequence, species richness is now higher but distinctness of the AMF communities could not be verified. Many OTUs were found in only one plant individual. With this study we want to test whether singleton OTUs form isolated associations with plants or if these OTUs are embedded in an overall network. Therefore, in addition to increased sampling, we applied network theories to assess nestedness and modularity of the AMF community. This is of interest, because it has been shown that a nested network structure contributes to high diversity [12] and robustness of community against extinction [20] and habitat loss [21]. We also assessed the level of specialisation between plants and AMF. We estimated AMF species richness and compared the similarity of AMF communities in the pristine forest and the reforestation sites. We analysed AMF communities of plant individuals and species and in particular for *Cedrela montana* Moritz ex Turcz. and *Tabebuia chrysantha* (Jacq.) G.Nicholson ( = *Handroanthus chrysanthus* (Jacq.) S.O.Grose) seedlings. These indigenous tree seedlings offer a good opportunity to study potential specificities of AMF. Our main goal was to investigate whether reforested and natural sites at Reserva Biológica San Francisco are similar and if plants and AMF in these sites are part of the same network. This is particularly interesting with respect to reforestation efforts. Therefore, the question whether the natural AMF community is still intact on the reforestation sites and could potentially provide a basis for regeneration of the natural plant vegetation was also of interest.

## Materials and Methods

### Ethics Statement

All necessary permits were obtained for the described field studies (Autorizacion de investigación científica by Ing. Carlos Espinosa G., Director Regional Provincal, Ministerio del Ambiente, Loja). The field study did not involve endangered or protected species.

### Study sites

The study was carried out in the natural forest and on managed sites in the area of Reserva Biológica San Francisco (RBSF) on the eastern slope of the Cordillera El Consuelo bordering the Podocarpus National Park, Zamora-Chinchipe Province (3°58′ S, 79°04′ W), southern Ecuador.

On the steep, north-facing slope covered with pristine forest, plots were established between 1900 and 2100 m asl [22]. A thinning treatment was conducted on some of the plots in June 2004. In February 2006, seedlings of native tree species were planted at these felling gaps as enrichment plantings [23].

The reforestation plots on the anthropogenically altered south-facing slope comprise abandoned pastures (R1), bracken fern areas (R2), shrub vegetation (R3), and a 30-year-old pine reforestation site (P), all located between 1900–2200 m asl. Each plot covers an area of four hectares.

The forest at pasture site R1 was cleared by slash and burn before 1976 [24], [25]. The site is now dominated by planted *Setaria sphacelata* (Schumach.) Stapf & C.E.Hubb. ex Moss which has been cultivated since the initial clearing. Before the reforestation experiment (in 2003), the site was actively used for milk production. The bracken site R2 was cleared in 1989 by slash and burn and since that was burnt at least four times [24], [25]. The last fire event occurred presumably in 2000. The shrub site R3 was cleared before 1986 and is now considered a secondary forest. Its vegetation is the result of natural succession without human intervention and by recurrent fires, the last fire occurring in 1993 [24], [25].

Seedlings of *Cedrela montana*, *Heliocarpus americanus* L., *Juglans neotropica* Diels, *Tabebuia chrysantha* (Jacq.) G. Nicholson, *Piptocoma discolor* (Kunth) Pruski and *Morella pubescens* (Humb. & Bonpl. ex Willd.) Wilbur were raised in the nursery with a mixture of highland black soil and bed sand. Forest humus was added as natural AMF inoculum [25]. Investigation of the AMF community in these nursery seedlings revealed a small number of widespread AMF OTUs, which occur also in reforestation plots [19]. After 6 months seedlings were planted in 2003 on the reforestation plots [26], [27].

### Sampling

We collected arbuscular mycorrhizae of 46 trees from the pristine forest, and additionally seedlings of the enrichment planting (*Cedrela montana, Nectandra membranacea* (Sw.) Griseb., *Tabebuia chrysantha*; 16 individuals) as well as naturally regenerated *Cedrela* seedlings (7 individuals). In total we analysed mycorrhizae of 61 plant individuals from the pristine forest (Tab. S1) sampled in 2001, 2003, 2004, and in 2010.

At the reforestation plots, mycorrhizae were sampled in 2004, 2006 and 2010 from 19 plant species (planted tree seedlings and neighbouring grasses, shrubs and trees), comprising 129 individuals (Tab. S1). All plants were determined to species, except for a few plants in the vicinity of seedlings.

Mycorrhizae were sampled by tracing single roots from the stem down to the fine roots. Mycorrhizae were cleaned under tap water on the same day and one part of each sample was fixed in 70% ethanol for light microscopy. The other part was dried in open tubes with an electric dryer at about 50°C for 24 hours, after which silica gel was added.

Many individuals were sampled from planted seedling species, especially *Cedrela* and *Tabebuia*, because they showed higher survival rates than the others [28], but from most tree species only one or two individuals could be sampled. All samples are listed in Tab.S1.

Colonization of the ethanol fixed mycorrhizae was examined using standard staining methods [29]. All samples were highly colonized by Glomeromycota and showed the typical features: arbuscules, coils, extra- and intracellular hyphae and vesicles.

### Molecular analyses

Mycobionts were investigated with molecular tools. Approximately 50 mg of dried mycorrhizae were ground with a carbide ball using a mixer mill (2×1.30 min, frequency 30/sec). Total DNA was isolated with the innuPREP Plant DNA Kit (Analytik Jena; Germany) and re-suspended in a final volume of 100 µl of elution buffer. The 18 S rDNA was amplified by using two rounds of PCR. The first PCR was performed with primers NS1/NS4 (GTA GTC ATA TGC TTG TCT C/CTT CCG TCA ATT CCT TTA AG, [30]) and for the nested PCR, primers AML1/AML2 (ATC AAC TTT CGA TGG TAG GAT AGA/GAA CCC AAA CAC TTT GGT TTC C, [31]) were used. Reaction volume for both PCR rounds was 25 µl with concentrations of 3 mM MgCl2, 200 µM for each dNTP (Life Technologies, Eggenstein, Germany), 0,5 µM for each primer (Biomers, Ulm, Germany), 1U Taq Polymerase (Life Technologies), amplification buffer (Life Technologies) and 0.2 µL 1% BSA (Bovine serum albumin; Sigma). A volume of 0.5 µl DNA extract was added to the first PCR and for the nested PCR, a volume of 1 µl of the PCR product was used as template. The annealing temperature for the first PCR was 40°C, and for the nested PCR we used a TOUCH program that reached an annealing temperature of 50°C starting from 60°C. Amplified PCR products were cloned with the Invitrogen TA Cloning Kit (Life Technologies) following the manufacturer's instructions, but using a third of the indicated volumes. Inserts were re-amplified from clones with primers M13F/M13R by picking eight bacterial clones with a toothpick and placing it directly to a PCR reaction mixture. Inserts were digested with restriction enzymes HinfI or AfaI. Digested products were examined on a 0.7% agarose gel. Two clones of the same RFLP pattern were cleaned either with ExoSAP-IT® (Affymetrix,Great Britain), PureLink PCR Purification Kit (Life Technologies) or peqGOLD Cycle-Pur Kit (Peqlab,Germany) and sequenced on an in-house ABI 3100 (Applied Biosystems, Germany) sequencer or by GATC Biotech (Konstanz, Germany). Whenever the two sequences were not identical, further clones of the same RFLP pattern were sequenced. In case analysis revealed more than three different sequences per PCR product, an additional eight clones were picked and analysed.

Sequences were edited with Sequencher (version 4.9, Gene Codes, Ann Arbor, Michigan), and a BLAST search was performed against the nucleotide sequence database (NCBl) [32]. In total, we obtained 1103 sequences; 75% of which belonged to Glomeromycota (825 sequences) and 25% were either not readable or from plants or animals. We checked for putative chimeric sequences with Bellerophon [33]. No chimeric sequences were detected in this study. When several inserts of a cloned PCR product belonged to the same OTU, only one sequence was included in the final data set, which consisted of 550 glomeromycotan sequences. Frequencies of OTUs were calculated as occurrences in different *individuals*. Newly generated glomeromycotan sequences (430) were deposited in GenBank under the accession numbers JX296680–JX297125 (Tab.S1). In addition, we included 120 sequences from previous studies [19], [34] in our data set.

Sequence alignments were done with MAFFT v6.847b (http://mafft.cbrc.jp/alignment/software/; strategy G-INS-i, [35]). We used PAUP version 4.0b10 [36] and *Endogone pisiformis* Link (Accession number X58724) as outgroup to estimate the phylogenetic relationships of all AMF sequences. Neighbor-Joining analyses using the BioNJ modification with Kimura 2-distances were carried out and combined with bootstrap analyses [37].

Operational Taxonomic Units (OTUs) were defined as surrogates for species on the basis of sequence similarity with OPTSIL [38]. We used intermediate linkage clustering and a cut-off value of≥97% and≥99% similarity on the 18 S rDNA. We chose a cut-off value of 97% to delineate OTUs because this value is commonly used. In addition, we analysed our data set with a cut-off value of 99% sequence similarity because the 18 S is relatively conserved and thus this delineation might correspond better to species than the 97% cut-off value. The linkage fraction was 0.5, which combines two clusters if the distances between 50% of the sequences in each cluster are equal or below the cut-off value [38]. OTU categorization was compared to clades in the NJ-tree. For the 99% similarity cut-off, most OTUs corresponded to monophyletic clades with a bootstrap support of 75% or higher (Fig.S1). Singleton-OTUs that were nested in monophyletic clades with high bootstrap support were sunken into the OTU of the corresponding clade.

We calculated a sample-based rarefaction accumulation curve with 95% confidence intervals, and estimated the total OTU richness with Chao2 and Jackknife2 using the software EstimateS 8.2.0 [39]. Sample randomization was performed without replacement. We calculated with EstimateS 8.2.0 the unbiased estimated Chao-Sørensen Index that includes unseen species [40], [41]. This approach has been shown to reduce substantially the negative bias that undermines the usefulness of traditional similarity indices, especially with incomplete sampling of communities [40]. Also we computed the Morisita-Horn-Index as an order 2 measure of beta diversity, which gives more weight to the most abundant species and is thus less biased by singletons and unseen species [42].

### Analyses of network structure

Networks as addressed here are by definition a set of nodes (species) connected through links (interactions). Mutualistic networks such as arbuscular mycorrhizae are two-mode networks because there are two types of nodes, arbuscular mycorrhizal fungi and plants; mutualistic interactions occur between but not within these node types.

We compiled three different data sets for each similarity cut-off (97% and 99%) to assess plant-AMF network structures: 1) all plant species and fungal OTUs from forest and reforestation plots together; 2) all plant species and OTUs from the reforestation plots only; 3) all plant species and OTUs from the forest only. We used the NODF (**n**estedness measure based on **o**verlap and **d**ecreasing **f**ills) measure implemented in Aninhado [43] to test for potential nestedness of plant-fungal networks at our study sites. Mycorrhiza network topology was based on qualitative links (presence/absence data). Therefore, binary matrices of in total six data sets were compiled. Two null models (ER: assigned interactions completely random with each cell in the matrix having equal probability; CE: randomization taking into account the average probability of presence in the respective row and column) were used to test for significance with 1000 randomizations for each data set. Connectance values and visualization of the networks were done with the R package “bipartite” (function “plotweb” for visualization and function “networklevel” for connectance values) [44].

In addition to nestedness, we tested for modularity in the network structure, using the program Netcarto [45]. Netcarto assesses compartments by simulated annealing to find the partition with the largest modularity. Significance is tested by comparing modularity values of the actual network and values obtained from similar-sized random networks [46]. Modularity was calculated only for data set 1 (all plant species and OTUs from forest and reforestation plots together) and plant individuals instead of species were used as entities. For the 99% similarity cut-off, iteration factor was set to 1.0, cooling factor to 0.995 and the analysis was run with 1000 randomizations. Modules of the network were visualized with the program Pajek [47]. For the 97% similarity cut-off, the network had a higher connectivity than for the 99% cut-off, so we chose an approach that is less computational intensive: iteration factor  =  0.4, cooling factor = 0.9, randomizations = 500. Significance of modularity was assessed by calculating a Z score based on the modularity of the actual AMF-plant network minus the mean modularity of the randomizations (M rand) and divided by the standard deviation of the randomizations (sigma M rand). The Z score was converted to a p–value.

Levels of specialisation between plants and AMF were assessed for the 99% similarity cut-off by calculating the indices H_2_′ and d' [48] implemented in the R package “bipartite” [44]. We used data set 1 (all plants and fungal OTUs from forest and reforestation sites together) and calculated an abundance matrix. Frequencies of OTUs were calculated as occurrences in different samples and not as frequencies of inserts in a cloned PCR product.

## Results

### Analysis of AMF sequences

**Figure 1:**
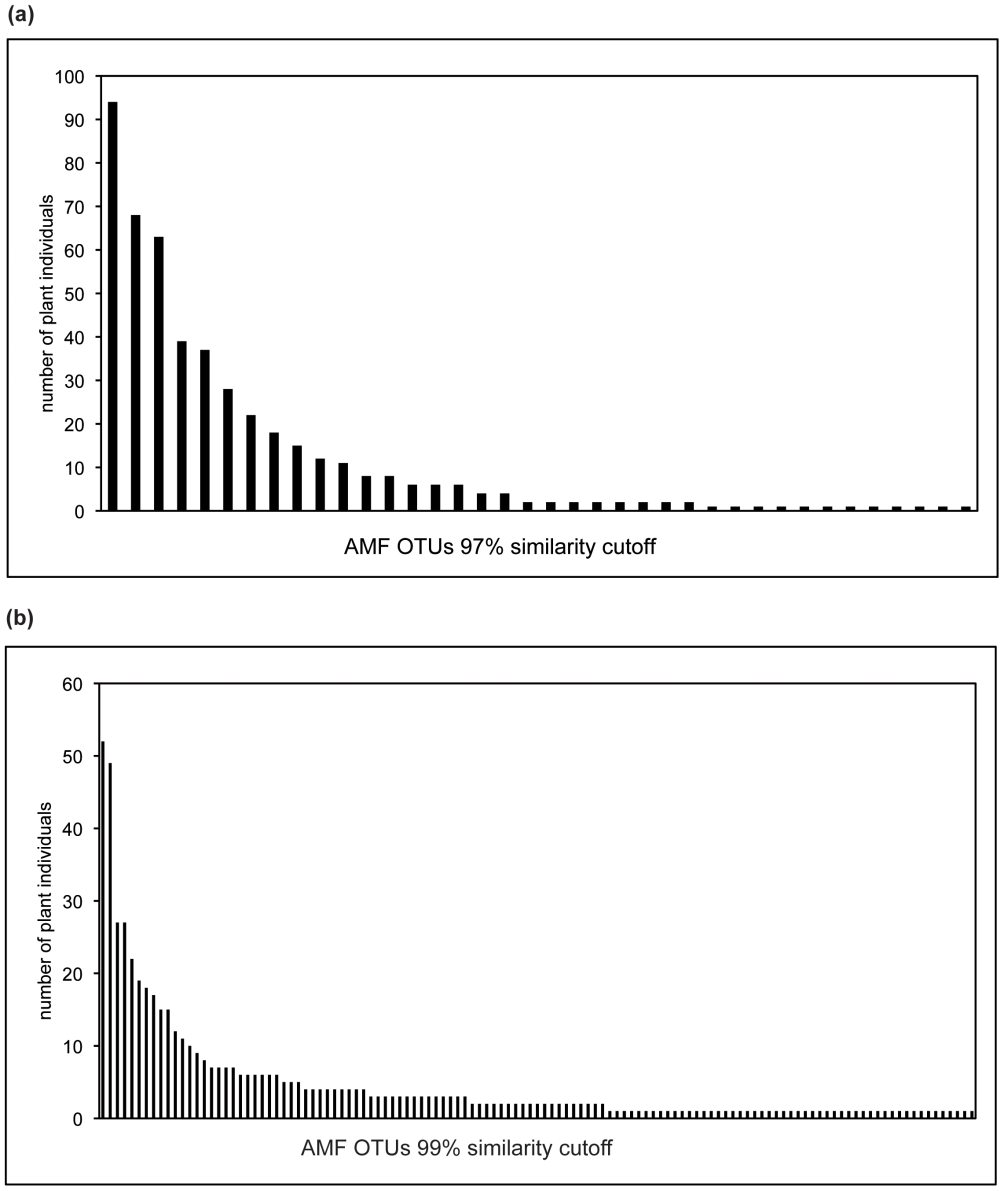
AMF OTU frequency distribution. The AMF community is characterized by few abundant and many rare OTUs. **a**. 97% similarity cut-off; **b**. 99% similarity cut-off.

From 213 individual plants we got 550 glomeromycotan sequences, most of them (430) are new for this study. On the basis of≥97% sequence similarity for part of the 18 S rDNA (about 800 bp) in combination with intermediate linkage clustering, 38 AMF-OTUs were identified with OPTSIL (Tab.S1, Fig.S1). On the basis of≥99% sequence similarity, we got 121 OTUs (Tab.S1, Fig.S1). In the following, the results of the 97% data set are written before a slash and the results of the 99% data set behind the slash. Most OTUs belonged to Glomerales (58/66%), followed by Diversisporales (18/22%), and Archaeosporales (24/12%) (Tab.S1, Fig.S1). Many of the OTUs were found only once or twice and only few OTUs were abundant in this area (Fig.1a,b, Tab.1). In the 99% similarity analysis, the highest frequency was found for OTU2 corresponding to *Rhizophagus intraradices/vesiculiferus/irregularis* occurring with 24% of the investigated plant individuals and representing 9.5% of all glomeromycotan sequences. For the 97% similarity analysis, OTU4 was the most frequent OTU. It was found with 43% of all investigated plant individuals and accounted for 17% of the glomeromycotan sequences. Twelve/fifty-one OTUs were singletons.

**Table 1:** Incidence of frequent OTUs in plant individuals and species, maximum numbers in bold.

OTU number	number of associated individuals	percentage of associated plant individuals	number of associated plant species
**97% similarity cut-off**			
2	18	8	9
4	**91**	**41**	23
5	22	10	8
8	39	18	21
12	68	31	21
14	37	17	16
15	63	29	**28**
21	11	5	6
22	27	12	13
23	12	5	7
30	15	7	8
**99% similarity cut-off**			
2	**52**	**24**	10
5	17	8	5
13	27	13	**18**
19	12	6	6
22	22	10	9
24	27	13	10
27	18	8	6
39	15	7	11
40	10	5	5
53	11	5	6
61	19	9	9
66	43	20	13
84	15	7	9

Frequent OTUs were associated with many plant species (Tab.1), with a maximum of 28 plant species for OTU15 at the 97% similarity level and 18 plant species for OTU13 at the 99% similarity level.

Rarefaction curves did not reach a stable state for both similarity cut-off values (Fig.S2a,b). The expected richness calculated with Chao2 and Jackknife2 estimated approximately 55 OTUs at the 97% similarity level and approximately 200 OTUs at the 99% similarity level for this site (Tab.S2).

### Comparison of AMF communities in pristine forest and reforestation plots

The richness of OTUs is similar for both sites: 28/73 OTUs were found on the reforestation plots and 28/81 OTUs in the pristine forest. On both sites Glomerales are clearly dominant (15/50 OTUs – reforestation site; 17/52 OTUs – pristine forest), followed by Diversisporales and Archaeosporales. Richness estimation reveals unsaturated rarefaction curves for both sites (Fig.S2c–f). Expected richness calculated with Chao2 and Jackknife2 is nearly identical with values of about 40/130 OTUs for each site (Tab.S2).

**Figure 2:**
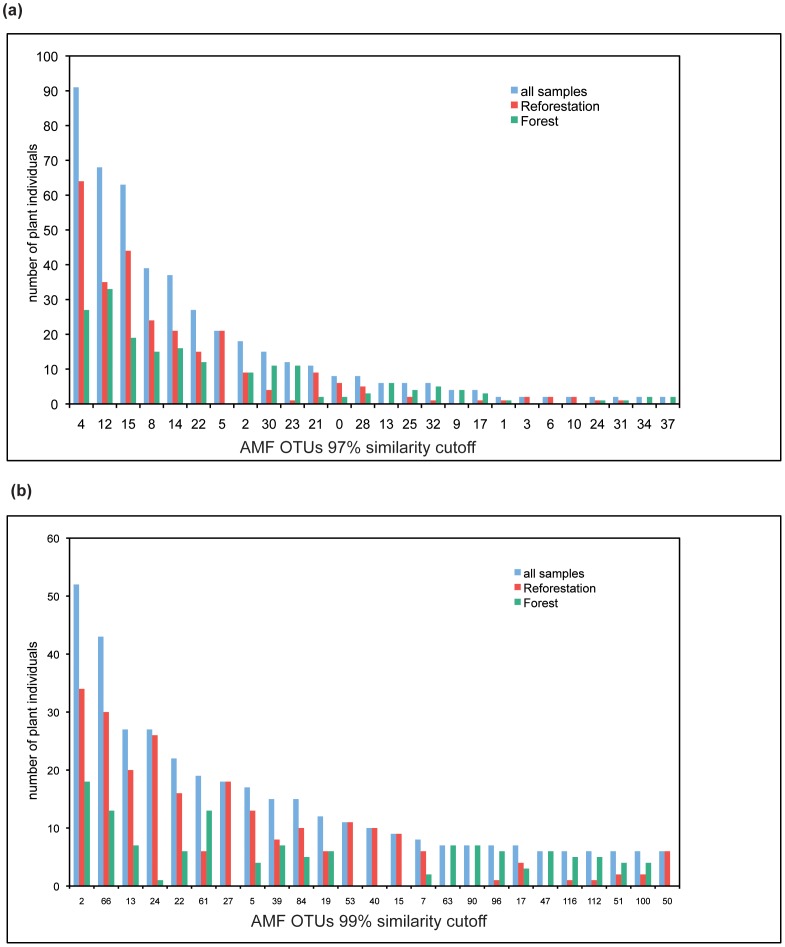
Frequencies of the numerous OTUs in the pristine forest (in green) and reforestation plots (in red). **a**. Frequencies of the 26 numerous OTUs at the 97% similarity cut-off; **b**. Frequencies of the 25 most numerous OTUs at the 99% similarity cut-off.

Most frequent OTUs occurred on both sites (Fig. 2a,b). OTU5 (97% similarity cut-off) was found only on the reforestation plots, OTU13 only in the pristine forest and several Archaeosporales-OTUs (OTU30,32,34,37) appear dominant in the pristine forest (Fig.2a). Similarities of reforestation and pristine forest AMF communities as estimated with Chao-Sørensen and indicated by Morisita-Horn Index were high (Tab. 2).

**Table 2:** Similarity indices of AMF communities (SD  =  Standard deviation), at the 97% and 99% similarity level.

	Chao-Sørensen estimator abundance based	Chao-Sørensen estimator Standard deviation abundance based	Morisita-Horn-Index
reforestation vs. forest 97%	**0.941**	0	**0.863**
reforestation vs. forest 99%	**0.708**	0.084	**0.616**
			
natural vs. planted on reforestation site 97%	**1**	0	**0.933**
natural vs. planted on reforestation site 99%	**0.91**	0.096	**0.736**
natural vs. planted *Cedrela* seedlings in pristine forest 97%	**0.735**	0	**0.713**
natural vs. planted *Cedrela* seedlings in pristine forest 99%	**0.748**	0.162	**0.656**

The number of OTUs for a single plant individual ranged from one to eight (97% similarity cut-off) and one to ten (99% similarity cut-off) for the forest and from one to six (97% similarity cut-off) and one to eight (99% similarity cut-off) for the reforestation plots. In most cases we detected one to four OTUs, while five or more OTUs were rare. The highest number of OTUs (8/10) was found in a naturally regenerated *Cedrela* seedling from the pristine forest.

Ninety-seven percent of all investigated plants were associated with at least one member of the Glomerales. Of these plants, 35% were also associated with members of the Diversisporales and/or Archaeosporales. The combined occurrence of Glomerales and members of Diversisporales and/or Archaeosporales in the same plant individual was higher in the forest (48%) than on the reforestation plots (27%).

Chao-Sørensen and Morisita-Horn Index indicated high similarities between AMF communities of naturally occurring plants (neighbouring grasses, shrubs, trees) and planted tree seedlings on the reforestation plots (Tab.2). The AMF communities of planted and naturally regenerated *Cedrela* seedlings from the pristine forest showed also high similarities (Tab.2).

### AMF community of *Cedrela*

In total, 30/74 OTUs were found in 61 *Cedrela* individuals. The rarefaction curves revealed no OTU saturation for *Cedrela* (Fig.S2g,h). Both richness indices, Chao2 and Jackknife2, revealed similar results and estimated about 55/150 OTUs for *Cedrela* (Tab.S2). On average, *Cedrela* individuals from the reforestation plots were associated with two/three OTUs and those from the pristine forest with approximately three/five OTUs. In the forest, planted and naturally occurring *Cedrela* seedlings were associated with approximately four/five OTUs; the AMF community of natural and planted *Cedrela* seedlings was similar (Tab.2).

AMF communities of *Cedrela* individuals ranged from one to eight/one to ten OTUs. *Cedrela* individuals with more than one OTU showed a variety of associated fungi (Tab. S3). However, one OTU was especially frequent. At the 97% similarity level it was OTU 4, which occurred with 70% of the investigated *Cedrela* individuals and only with 32% of the remaining plant individuals. At the 99% similarity level it was OTU2 occurring in 67% of all investigated individuals of *Cedrela* from the forest and in about half (49%) of the *Cedrela* plants from the reforestation plots. By comparison, OTU2 was associated with 9% of all other forest plants and with 15% of the remaining plants from the reforestation plots.

### AMF community of *Tabebuia*

For *Tabebuia*, 16/38 OTUs were found in total. The rarefaction curves did not reach their asymptotes (Fig.S2i,j). Both richness indices, Chao2 and Jackknife2, estimated values of about 22/60 OTUs for *Tabebuia* (Tab.S2). *Tabebuia* individuals growing in the reforestation plots showed two OTUs on average. In the forest, *Tabebuia* plants were associated with about two/four different OTUs. OTU distribution among *Tabebuia* individuals was unpredictable and showed a great diversity (Tab.S4), but with a high frequency of OTU66 at the 99% similarity level: 37,5% of the investigated individuals in the forest and 48% on the reforestation plots were associated with OTU66. From the other plant individuals, 13% of the forest and 15% from the reforestation plots occurred with OTU66. The most frequent OTU, OTU2, was not detected in *Tabebuia*. For the 97% similarity cut-off, OTU12 was the dominant OTU of *Tabebuia* occurring with 51% of the investigated *Tabebuia* individuals.

### Network analysis

**Figure 3:**
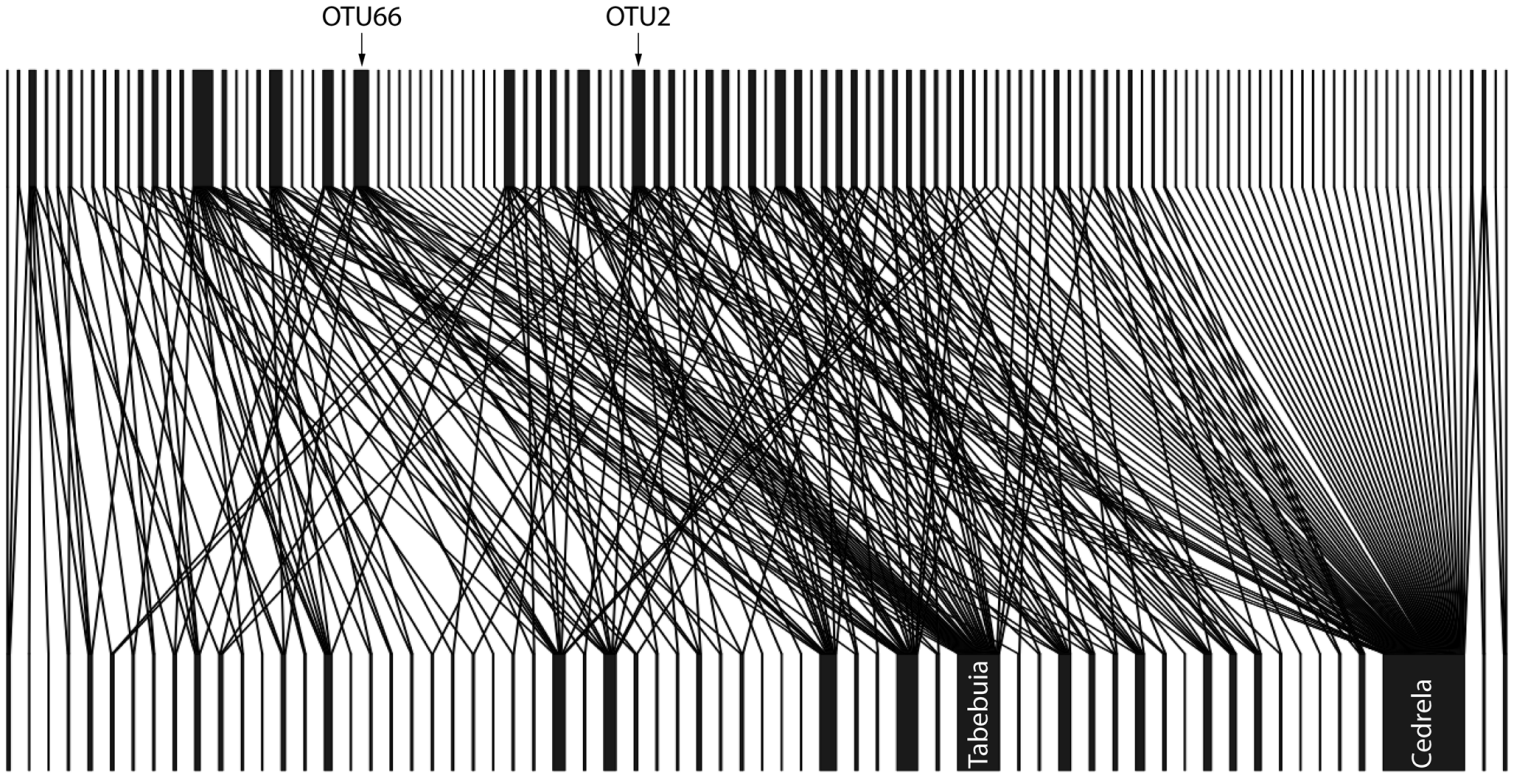
Network structure of plants and AMF from the pristine forest and reforestation plots at RBSF (99% similarity cut-off). Bars on top display fungal OTUs, while plant species are indicated on the bottom of the image. The network between plants and AM fungi in the study area is significantly nested.

Nestedness values range from 0 to 100, where 100 indicates complete nestedness and 0 non-nested structures. All tested data sets show that networks between plants and AMF in the study area are significantly more nested than expected by chance (Tab.3, Fig.3). The nestedess values are lower for the 99% similarity cut-off than for the 97% cut-off. The same is true for the connectance values. The highest NODF values (59.5/37.2) were found at the reforestation plots (Tab.3). For the data set including forest and reforestation, plant species had a higher nestedness than the fungi (Tab.3). On the reforestation plots the values were similar for plants and fungi.

**Table 3:** Network metrics at the 97% and 99% similarity level.

Data set	NODF fungi	NODF plants	NODF total	P ER	P Ce	Connectance values
F+Ref 97%	33,69	55,28	35,85	0	0	0.12
F+Ref 99%	13,06	31,24	27,38	0	0	0.04
Ref 97%	59,01	59,84	59,46	0	0	0.23
Ref 99%	40,01	37,22	37,42	0	0	0.11
F 97%	23,83	38,12	27,78	0	0	0.10
F 99%	6,84	15,9	13,71	0	0	0.05

(Abbreviations: F  =  forest, Ref  =  reforestation)

### Specificity and modularity

The H_2_′ index describes the deviation from a completely neutral configuration of associations with values ranging from 0 (no specificity) to 1 (complete specificity). This index resulted in a value of 0.198, suggesting no specificity of interactions.

We tested for modularity in the complex AMF-plant network on the basis of plant individuals in order to investigate whether habitat or plant species drive AMF communities in the study area. Values of modularity (M) range from 0 to 1, with values>0.3 indicating modularity [46]. At the 97% similarity level, no modularity was found (M = 0.177, p-value = 0.4). At the 99% similarity level, however, the network was significantly modular (M = 0.611, p-value<0.0001) and partitioned into 12 modules (Fig.S3, Tab.S5). Repeatedly sampled species are not united in their own modules, but are placed in different modules (Tab.S5). All modules, with the exception of module 13, are embedded in the overall network, which is indicated by many links across different modules (Fig.S3). Modules 1, 2, 6, 9 and 11 include plant individuals from different plots, whereas modules 3, 4, 5, 7, 8 and 12 consist primarily of plants from only one plot each (Fig.S3; Tab.S5). Module 4 shows the highest percentage (76%) of within-links (Tab.S6) and consists only of plants and fungi from the forest (with exception of OTU16, which is also found once on reforestation plots). Five modules have percentages of within-links ranging from 59% to 63% (modules 3, 7, 9, 11 and 12) and five modules (1, 2, 5, 6, 8) have values around 50% (Tab.S6).

## Discussion

### Richness of AMF OTUs for sites, species and individuals

We found a high richness of Glomeromycota in the tropical mountain rainforest, on the reforestation plots and within individual plant species. In order to assess this richness, comparison to other sites is necessary. However, even when using the same similarity cut-off values, comparison among different studies is difficult because different sampling methodologies, sequencing methods and clustering procedures are used. Nevertheless, when comparing our results to those found in 111 other studies (Tab. 4, [49]), the number of OTUs found in our study is exceptionally high. High numbers of AMF OTUs were also reported from large scale parallel 454 sequencing studies in boreo-nemoral forests ([50] 47 AMF taxa) and in a semi-natural grassland ([51] 32 AMF OTUs).

**Table 4:** AMF OTU richness on the 18 S region.

	97% similarity cut-off	99% similarity cut-off
pristine forest + reforestation	38	121
pristine forest	28	81
reforestation	28	73
*Cedrela*	30	74
*Tabebuia*	16	38
average, calculated from 111 sites [49]	15,39	17,57

The fungal species richness is in good accordance with the outstanding diversity of vascular plants (1,208 spermatophytes; [52]) corroborating the suggestion of Fitter [53] that fungal and plant species richness are linked. After analyzing over 200 plant individuals, the rarefaction curves are still unsaturated, even for the two intensively sampled plant species *Cedrela montana* and *Tabebuia chrysantha*. This is in contrast to most other AMF studies where rarefaction curves reached either a saturated level [50], [54], [55] or at least captured a large portion of the AMF diversity [51], [56], [57] even with fewer samples. For *Cedrela montana* alone, we found 30/74 OTUs (97/99% similarity level). Moora et al. [55] recorded 73 AMF taxa for *Trachycarpus fortunei* (Hook.) H.Wendl. sampling at 14 sites in Europe and Asia. Diversity of AMF for *Tabebuia* seems to be lower than for *Cedrela*. We detected 16/38 OTUs in *Tabebuia* and richness estimation with Chao2 and Jackknife2 calculated half the number of OTUs for *Tabebuia* as for *Cedrela*. Nevertheless, AMF diversity in *Tabebuia* is still high.

With every new sample, the number of OTUs increased. It remains to be seen whether all plant species in the tropical mountain rain forest associate with such a high diversity of fungi, but based on our findings it is likely. Our results suggest that many plant species and fungal OTUs in the mountain rain forest are interaction generalists [58]. However, some fungal species might be more specific than others. To detect this specificity, a large number of individuals from the same plant species need to be collected - a condition that is difficult to find in tropical mountain rain forests, because many plant species occur only in very low numbers [22].

In our study area, the most frequent fungus of the AMF community is OTU 4 at the 97% similarity level and respectively OTU2 at the 99% similarity level. However, OTU 4/2 represents only 17%/9.5% of total abundance within the community, a value that is much lower as reported for other dominant Glomeromycota [59]. Dumbrell and coauthors [59] postulated that AM fungal communities are typically dominated by a single taxon, representing on average 40% of total abundance. The discrepancy in percentages for abundant AMF OTUs in this and the Dumbrell study may be influenced by different evaluation practices. Here, we calculated OTU abundances as occurrences in different plant individuals and not as number of clones of the same sample. Clone frequency can be biased towards a specific fungus rather than show true abundance of a certain AMF by favourable PCR conditions and therefore can result in much higher values.

The BLAST search revealed that the abundant OTU2 (99% similarity cut-off) has a high similarity to *Rhizophagus intraradices/vesiculiferus/irregularis* (taxonomy after [60]). Many other studies [51], [61], [62], [63], [64], [65] report that this cosmopolitan species complex is very abundant at other sites. Though, it is likely that the *Rhizophagus intraradices/vesiculiferus/irregularis* complex is not globally distributed, but rather consists of different strains with a restricted distribution. However, phylogenetic analyses could not fully resolve this lineage so far [66].

The five most frequent OTUs in our study represent 50/31.5% of all sequences in the data set. Ninety-nine/sixty-two percent of all investigated plant individuals are associated with one or several of these fungi. Such a high level of generalisation of the plant-AMF community may have network-stabilizing functions and serve as a buffer against coextinction. Other benefits of having different symbiotic partners and the question why some fungi are rare and others frequent are as yet unexplored. Differences in propagation or growth rates may have an influence, but are difficult to demonstrate in field studies. Kiers and coauthors [67] experimentally showed that plants are able to select those fungi that supply the most phosphate and reward them with more carbohydrates in return. Thus, plants and fungi with the most efficient associations may eventually become the most frequent. On the other hand, different species may have different requirements, which could be the reason why dominant fungi differ in *Cedrela* and *Tabebuia*.

### Specificity of AMF OTUs for species and sites

In general, we did not identify plant species with specific OTUs but we did observe preferences within the multi-sampled *Cedrela* and *Tabebuia*. On average, *Cedrela* associated more often with OTU2 and *Tabebuia* with OTU66 (similarity cut-off 99%). However, OTUs 2 and 66 are not species specific – they occur with many other plant species at our sampling sites and worldwide. We also found no indication for specificity of AMF, even at the 99% similarity level. Frequently sampled OTUs always occurred on several different plant species. Low specificity of plants and fungi in the study area is also supported by low H_2_′ and d_2_′ values.

Only few OTUs were found either in the pristine forest or on reforestation plots and further sampling need to show if this holds true.

### Network analysis

Networks of AMF and plants in our study show significant nested structures. In our study, NODF values were lowest for the pristine forest. Low network connectivity (or matrix fill) is likely the reason for these low values, because Almeida-Neto and coauthors [68] found a correlation between low connectivity and low nestedness values. These lower values might also be due to the fact that in the species-rich, pristine forest more as twice as much plant species were investigated as at the reforestation site. Furthermore, the higher values of the reforestation site may be based on the high sampling numbers of *Cedrela*, which shows a very species-rich AMF community. It is still under discussion which sampling strategy is more representative data set[16], [69]. Thus, we do not want to over-interpret the different NODF values between pristine forest and reforestation. We only want to point out that at both sites rare AMF OTUs are integrated in the overall network. Higher plant nestedness found in the pristine forest was also verified for the plant-AMF community in a semi-arid region in Mexico [16]. Our analyses show that rare AMF OTUs are associated with plant species that again are allied with other, frequent OTUs. Rare species may enhance the stability of a complex community by providing characteristics favoured in a changing environment. Thus, rare species can turn into frequent species depending on environmental conditions. Complementarity and convergence in evolution of bidirectional nutrient exchange and recognition processes favour generalist partnerships in mycorrhizae and maintain phylogenetically closely related species in the networks [18]. This may explain the great diversity of closely related Glomerales in the same habitat.

### Modularity

Modularity in our AMF-plant network structure was only found at the 99% similarity level (M = 0.611, p-value<0.0001). It is likely that the non-modularity of the 97% similarity network is due to high nestedness and connectivity because these values were considerably higher than for the network at the 99% similarity level. Fortuna and coauthors [9] found a correlation between low nestedness and high modularity, when connectivity values are low. However, the differing results could also have been influenced by the different parameters used.

We could not detect a single main driver for the modules detected at the 99% similarity level. Some modules appear to be determined by the environment and others by potential preferences for specific partners. However, for many modules no obvious reason could be detected. Most modules were well-embedded into the network and showed as many links to other modules as to nodes within the same module. Considering the fact that the rarefaction curves are unsaturated, even more links between the modules are likely to occur and we would expect that modularity would rather decrease than increase.

### Comparison pristine forest – reforestation sites

Arbuscular mycorrhizal fungal communities of the pristine forest and reforestation plots share many features: Similar richness and diversity, similar expected richness, similar numbers of OTUs per plant individual, and most of all a nested network structure and high similarity of OTU composition. This suggests that in our study area, mycobionts of the forest vegetation have been adopted by the new flora. Thus, a similar AMF community in composition and richness could be maintained over 36 years of anthropogenic use and several fire events. Size of the disturbed sites might play a critical role for maintenance of AMF communities. Disturbed sites in our study were intercepted by ravines with more or less natural forest vegetation. In addition, the pristine forest was in close reach. Therefore, input of AMF inoculum from the adjacent forests to the disturbed sites is another plausible explanation on how high diversity and similar composition of AMF could be maintained.

The results indicate that in our study sites, regeneration potential of the natural forest vegetation is not inhibited by a lack of appropriate mycobionts. The highly diverse flora on the human influenced sites most likely serves as a provider of the diverse generalist mycobionts. Forest management by planting native tree seedlings can foster the ongoing process of rehabilitation. Further research is being carried out to examine and fine-tune these hypotheses.

## Supporting Information

Figure S1 **Phylogenetic relationships of Glomeromycota sequences obtained with primers AML1 and AML2 from mycorrhizae in a tropical mountain rain forest (in green letters) and neighboring reforestation sites (in red letters) in South Ecuador.** The names of each sequence start with either the clone-number or the Genbank accession number, the site (**F** pristine forest, **Fp** planted seedling in pristine forest, **Fn** naturally regenerated seedling in pristine forest, **R1** reforestation site - pasture plot, **R2** reforestation site - fern plot, **R3** reforestation site - shrub plot, **P** reforestation site - gap in pine forest) and the associated plant genus. More details on the sequences are given in Tab.S1. The tree was rooted with *Endogone pisiformis*. Numbers on branches show bootstrap values. Corresponding OTU numbers are shown for the 97% similarity level (in blue) and for the 99% similarity level (in black).
(TIF)

Figure S2 Rarefaction curves for AMF OTUs. Rarefaction curves of the different data sets (a–j) did not reach a stable state. (Sobs  =  species observed, LB  =  lower bound, UB  =  upper bound). **a**) Samples of pristine forest and reforestation plots, 97% similarity cut-off; **b**) Samples of pristine forest and reforestation plots, 99% similarity cut-off; **c**) Samples of pristine forest, 97% similarity cut-off; **d**) Samples of pristine forest, 99% similarity cut-off; **e**) Samples of reforestation plots, 97% similarity cut-off; **f**) Samples of reforestation plots, 99% similarity cut-off; **g**) Samples of *Cedrela* from pristine forest and reforestation plots, 97% similarity cut-off; **h**) Samples of *Cedrela* from pristine forest and reforestation plots, 97% similarity cut-off; **i**) Samples of *Tabebuia* from pristine forest and reforestation plots, 97% similarity cut-off; **j**) Samples of *Tabebuia* from pristine forest and reforestation plots, 99% similarity cut-off.
(TIF)

Figure S3 **Modules of plant-AMF relationships in a nested network (99% similarity cut-off).** Numbers indicate module ID and percentages give the number of within-links in comparison to among-links. The higher the percentage, the more isolated is the module. All modules, with the exception of module 13, are embedded in the overall network, which is indicated by many links across different modules.
(TIF)

Table S1 List of all sequences with OTU numbers for the 97% similarity cut-off and the 99% similarity cut-off as well as Genbank accession number, associated plant species (numbered if there are several sampled individuals), site, order of Glomeromycota and labelling in Fig.S1.
(XLS)

Table S2 OTU richness estimation for the following data sets: all samples combined, pristine forest only, reforestation plots only, *Cedrela* only and *Tabebuia* only.
(XLS)

Table S3 AMF OTU composition for individual *Cedrela* seedlings.
(XLS)

Table S4 AMF OTU composition for individual *Tabebuia* seedlings.
(XLS)

Table S5 Detailed information of module composition.
(XLS)

Table S6 Module metrics.
(XLS)
