# Supplementary figures and images for: Reforestation Sites Show Similar and Nested AMF Communities to an Adjacent Pristine Forest in a Tropical Mountain Area of South Ecuador
Source: PLoS One. 2013 May 6;8(5):e63524. doi: 10.1371/journal.pone.0063524 (PMC3646028; doi:10.1371/journal.pone.0063524)

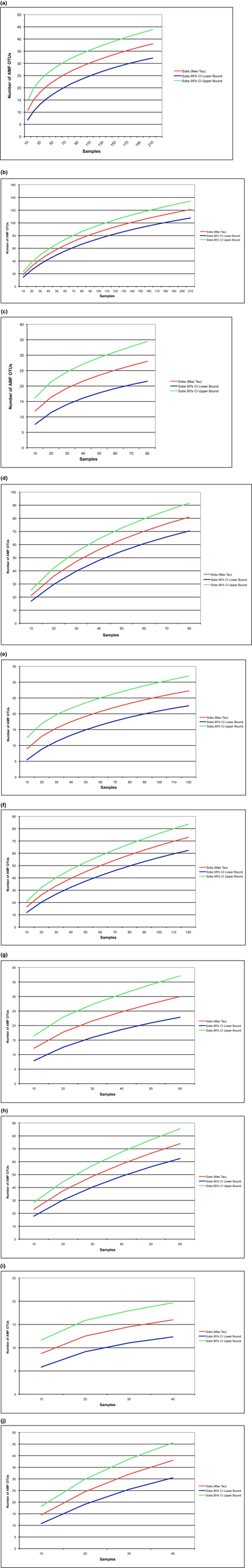

Supplement: Figure S2 — Rarefaction curves for AMF OTUs. Rarefaction curves of the different data sets (a–j) did not reach a stable state. (Sobs = species observed, LB = lower bound, UB = upper bound). a) Samples of pristine forest and reforestation plots, 97% similarity cut-off; b) Samples of pristine forest and reforestation plots, 99% similarity cut-off; c) Samples of pristine forest, 97% similarity cut-off; d) Samples of pristine forest, 99% similarity cut-off; e) Samples of reforestation plots, 97% similarity cut-off; f) Samples of reforestation plots, 99% similarity cut-off; g) Samples of Cedrela from pristine forest and reforestation plots, 97% similarity cut-off; h) Samples of Cedrela from pristine forest and reforestation plots, 97% similarity cut-off; i) Samples of Tabebuia from pristine forest and reforestation plots, 97% similarity cut-off; j) Samples of Tabebuia from pristine forest and reforestation plots, 99% similarity cut-off. (TIF) [file pone.0063524.s002.tif]

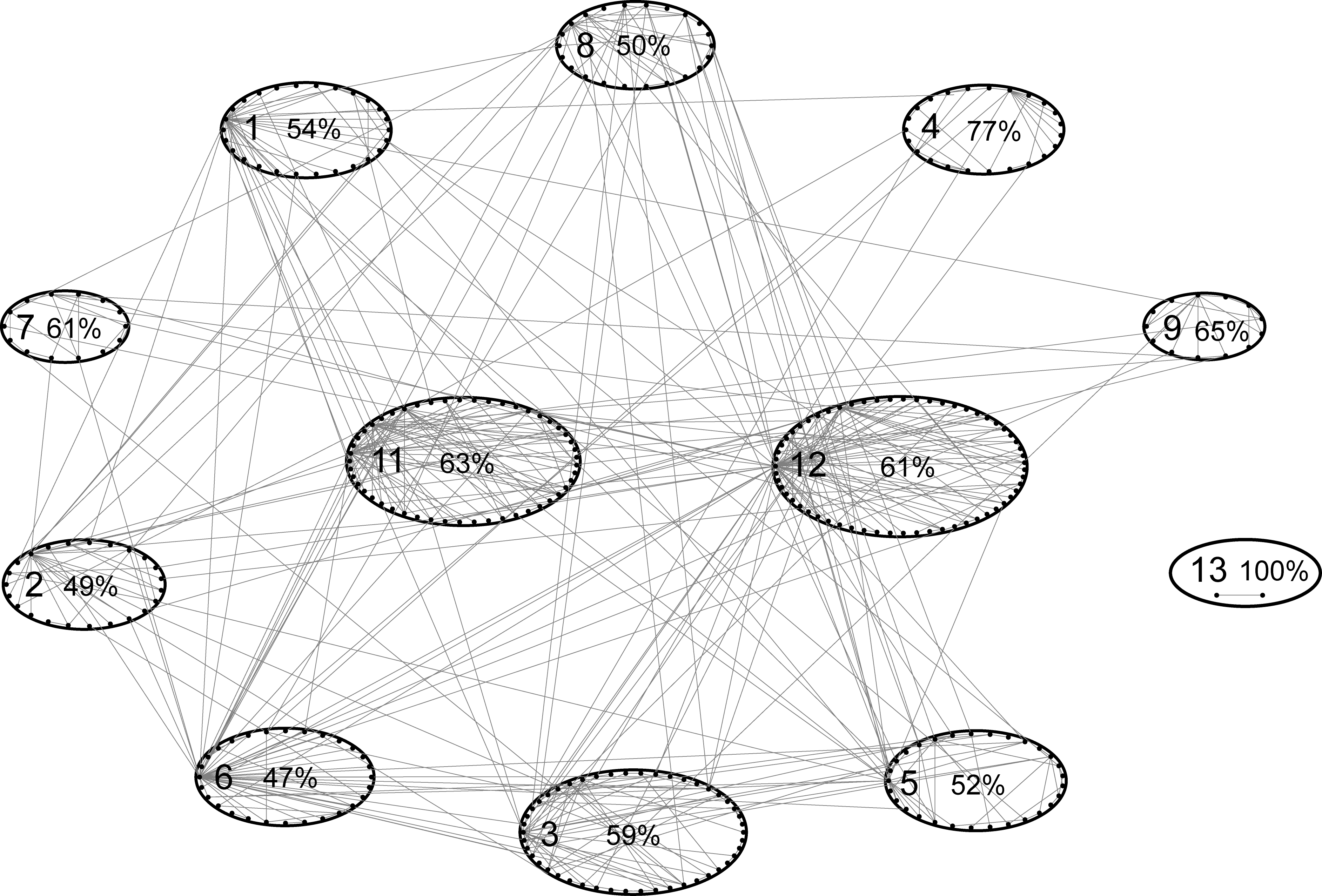

Supplement: Figure S3 — Modules of plant-AMF relationships in a nested network (99% similarity cut-off). Numbers indicate module ID and percentages give the number of within-links in comparison to among-links. The higher the percentage, the more isolated is the module. All modules, with the exception of module 13, are embedded in the overall network, which is indicated by many links across different modules. (TIF) [file pone.0063524.s003.tif]
